# Symptoms of Anxiety and Depression in Young Athletes Using the Hospital Anxiety and Depression Scale

**DOI:** 10.3389/fphys.2018.00182

**Published:** 2018-03-07

**Authors:** Stephanie Weber, Christian Puta, Melanie Lesinski, Brunhild Gabriel, Thomas Steidten, Karl-Jürgen Bär, Marco Herbsleb, Urs Granacher, Holger H. W. Gabriel

**Affiliations:** ^1^Department of Sports Medicine and Health Promotion, Friedrich Schiller University Jena, Jena, Germany; ^2^Division of Training and Movement Sciences, Research Focus Cognition Sciences, University of Potsdam, Potsdam, Germany; ^3^Department of Psychiatry and Psychotherapy, University Hospital Jena, Jena, Germany

**Keywords:** youth athletes, anxiety, depression, gender differences, late childhood, adolescents

## Abstract

Elite young athletes have to cope with multiple psychological demands such as training volume, mental and physical fatigue, spatial separation of family and friends or time management problems may lead to reduced mental and physical recovery. While normative data regarding symptoms of anxiety and depression for the general population is available (Hinz and Brähler, 2011), hardly any information exists for adolescents in general and young athletes in particular. Therefore, the aim of this study was to assess overall symptoms of anxiety and depression in young athletes as well as possible sex differences. The survey was carried out within the scope of the study “Resistance Training in Young Athletes” (KINGS-Study). Between August 2015 and September 2016, 326 young athletes aged (mean ± SD) 14.3 ± 1.6 years completed the Hospital Anxiety and Depression Scale (HAD Scale). Regarding the analysis of age on the anxiety and depression subscales, age groups were classified as follows: late childhood (12–14 years) and late adolescence (15–18 years). The participating young athletes were recruited from Olympic weight lifting, handball, judo, track and field athletics, boxing, soccer, gymnastics, ice speed skating, volleyball, and rowing. Anxiety and depression scores were (mean ± SD) 4.3 ± 3.0 and 2.8 ± 2.9, respectively. In the subscale anxiety, 22 cases (6.7%) showed subclinical scores and 11 cases (3.4%) showed clinical relevant score values. When analyzing the depression subscale, 31 cases (9.5%) showed subclinical score values and 12 cases (3.7%) showed clinically important values. No significant differences were found between male and female athletes (*p* ≥ 0.05). No statistically significant differences in the HADS scores were found between male athletes of late childhood and late adolescents (*p* ≥ 0.05). To the best of our knowledge, this is the first report describing questionnaire based indicators of symptoms of anxiety and depression in young athletes. Our data implies the need for sports medical as well as sports psychiatric support for young athletes. In addition, our results demonstrated that the chronological classification concerning age did not influence HAD Scale outcomes. Future research should focus on sports medical and sports psychiatric interventional approaches with the goal to prevent anxiety and depression as well as teaching coping strategies to young athletes.

## Introduction

During stressful situations, the body is threatened by external or internal forces that may lead to an alteration of its homeostasis. The adaptive changes, which occur in the body during stress, can either be behavioral or physical. Physiologically, stress stimulates the activation of the sympathetic nervous system and the hypothalamic-pituitary-adrenal axis (Messina et al., 2016). Psychologically, increased stress may lead to the development of symptoms of anxiety and depression (Mineka and Zinbarg, 2006).

The development of psychological distress and the prevalence of anxiety and depression in the athletic population is of interest to athletes, coaches, parents, teachers, and the scientific community, and has recently gained increasing awareness by the public in general. Current research lacks a precise description of prevalence rates of anxiety disorder or major depressive disorder in high performance athletes. Most studies used questionnaires leading to both, over and under estimation of occurrence rates of anxiety or depressive symptoms (Storch et al., 2005; Yang et al., 2007; Gulliver et al., 2014; Gouttebarge et al., 2015; Junge and Feddermann-Demont, 2015). Thus, descriptions of anxiety symptoms in the adult athlete population range from 7.1 to 26% (Gulliver et al., 2014; Gouttebarge et al., 2015) and symptoms of depression from 10.3 to 27.2% (Gulliver et al., 2014; Junge and Feddermann-Demont, 2015). In contrast, intercollegiate student-athletes have higher anxiety symptom rates of up to 37% (Storch et al., 2005) but similar rates of depressive symptoms (21%) (Yang et al., 2007). These variabilities might be explained by methodological differences such as application of different questionnaire, or differences in time of testing during a training season for example training or competition phase. In addition, assessment time of the day, including before or after a training session could explain the differences (Hines, 2004; Moskowitz and Young, 2006). Further, it is important to stress, that the use of questionnaires is very susceptible to be biased by confounding covariates (Luppino et al., 2010) and is by no means sufficient to establish a clinical diagnosis. However, likely reasons for these high rates are the elevated risk of injuries, performance plateaus or decrements or an approaching retirement form elite sports (Rice et al., 2016). This could explain the 37% prevalence rate of anxiety symptoms in college student-athletes. Furthermore, it has been suggested that transition stages in the athletic career are accompanied by increased stress levels and emotional imbalances. All these factors contribute to the higher anxiety symptom rates described in college student-athletes.

With regards to the young athlete population, limited research regarding anxiety and depressive symptoms is available. For instance, Brand et al. (2013) investigated psychological symptoms in elite student athletes compared to non-athletes aged 12–15 years. Findings show higher anxiety and depressive symptom frequencies for female compared to male students regardless of their athletic status. In addition, anxiety and depressive symptom scores were higher in the student athletes compared with their non-athletic peers. However, this finding was only significant for the anxiety level in female participants. Further, Nixdorf et al. (2016) found higher levels of depressive symptoms in athletes (mean age: 14.96 ± 1.56) participating in individual sports compared to team sports.

For young athletes, the time of adolescence can be regarded as a transition stage. Transition stages in the athletic career are characterized by changes on a psychosocial, academic vocational, and psychological level (Wylleman et al., 2004). On a psychosocial level, the athlete changes from relying entirely on his/her parents, siblings, and peers, to peer to peer relationships and coach-athlete relationship. On an academic vocational level, young athletes are faced with the challenges of entering secondary education, whilst on a psychological level having to deal with the changes that occur in the body during adolescence. Finally, on an athletic level, young athletes are faced with the challenges of developing sport-specific skills and techniques, increased training volumes and intensities, and higher competition frequencies (Wylleman et al., 2004). These multiple demands might be associated with an increased occurrence rate of anxiety or depressive symptoms. However, there is far more research necessary.

Whilst the research of Brand et al. (2013) and Nixdorf et al. (2016) clearly show the presence of symptoms of anxiety and depression in young athletes, they do not indicate how severe these symptoms are. However, it is of high relevance for athletes, parents, coaches, and sport psychologists to elucidate the clinical relevance of these symptoms. One way to measure symptoms of anxiety and depression and their severity is the use of the Hospital Anxiety and Depression Scale (HAD Scale). The HAD Scale is a questionnaire, which has been developed to detect the overall state and severity of anxiety and depression (Zigmond and Snaith, 1983). Since its development, various international versions and reference values have been generated for a variety of patient groups in the adult population (Herrmann, 1997). In addition, the HAD Scale has been validated for the use in the adolescent population (White et al., 1999). However, limited data regarding normative values are available. Furthermore, to the best of our knowledge the athletic population has not been studied yet.

Therefore, the aim of this study was to provide an overview of symptoms of anxiety and depression in young athletes using the HAD Scale. The following research questions were specifically addressed:

1. How are the symptoms of anxiety and depression in young athletes distributed?
2. How severe are clinically relevant symptoms of anxiety and depression?
3. Are there any age and sex differences for symptoms of anxiety and depression in young athletes?

## Materials and methods

### Participants

The cross-sectional survey was carried out within the scope of the study “Resistance Training in Youth Athletes” (KINGS-Study). Between August 2015 and June 2017, 326 young athletes aged 12–18 years (mean ± SD 14.3 ± 1.6 years) were asked to complete the HAD Scale. Of these 155 were male and 171 female athletes. The participating young athletes were recruited from handball (80), volleyball (43), judo (41), canoeing (39), track and field athletics (32), soccer (25), rowing (21), ski jumping (15), Olympic weight lifting (10), boxing (10), ice speed skating (8), and gymnastics (2).

The study was carried out in accordance with the Declaration of Helsinki and was approved by the Ethics committee of the Friedrich Schiller University Jena (4584-10/15), Germany. All subjects and their legal representatives gave their written informed consent after having been thoroughly informed about the nature and course of the experiment.

### Schooling background

Regarding the schooling background, all participating athletes except ski jumping athletes were from *Eliteschulen des Sports* (elite sport schools). Elite sport schools are facilities in which highly gifted and/or talented athletes are given an opportunity to develop and maximize their sporting talent, whilst maintaining school education (Emrich et al., 2009). The ski jumping athletes were partly from elite sport schools and partly from a general Gymnasium (high-school).

### The hospital anxiety and depression scale

The HAD Scale was originally developed and validated by Zigmond and Snaith (1983) with the intention to detect states of depression and anxiety in adults aged 16–65 years. It contains an anxiety (HADS-A) and a depression (HADS-D) subscale each consisting of 7 items, rated on a four point Likert scale (0–3), therefore a maximum count of 21 points per subscale is possible. The questions are designed to focus solely on psychiatric symptoms by excluding questions related to physical illness such as dizziness or headaches, thereby excluding somatic components (Zigmond and Snaith, 1983). The questionnaire is designed to assess the participants' state over the past 2 weeks. In the studies on the adult population, the HAD Scale shows good internal consistency, good diagnostic qualities and case-finding properties (Bjelland et al., 2002; Brennan et al., 2010).

Original cut-offs for anxiety and depression have been defined as 0–7 no case, 8–10 doubtful case, and 11–21 case (Table 1). White et al. (1999) validated the HAD Scale for the use in the adolescent population (12–16 years) and suggested different cut-offs for the interpretation of severity. For HADS-A 0–8: no case, 9–11 possible case, 11–21 probable case and for HADS-D: 0–6 no case, 7–9 possible case, 10–21 probable case. It was argued that a higher threshold value for depression (10+) and anxiety (12+) would minimize false positive diagnostics, whereas a lower cut-off for depression (7) and anxiety (9) would minimize false negative diagnostics. Research on the adolescent population showed sufficient validity, psychometric properties and internal consistency of the HAD Scale (White et al., 1999; Chan et al., 2010). Since the HAD Scale is a questionnaire developed to detect clinical forms of anxiety and depression, the cut-off scores could equally be named as subclinical and clinical values.

**Table 1:** Anxiety and depression cut-offs for the adult and adolescent population.

	**No cases**	**Doubtful cases**	**cases**
Zigmond and Snaith, 1983	**Anxiety and depression**		
	0–7	8–10	11–21
White et al., 1999	**Anxiety**		
	0–8	9–11	>11
	**Depression**		
	0–6	7–9	>9

### Data processing and statistical analysis

**Figure 1:**
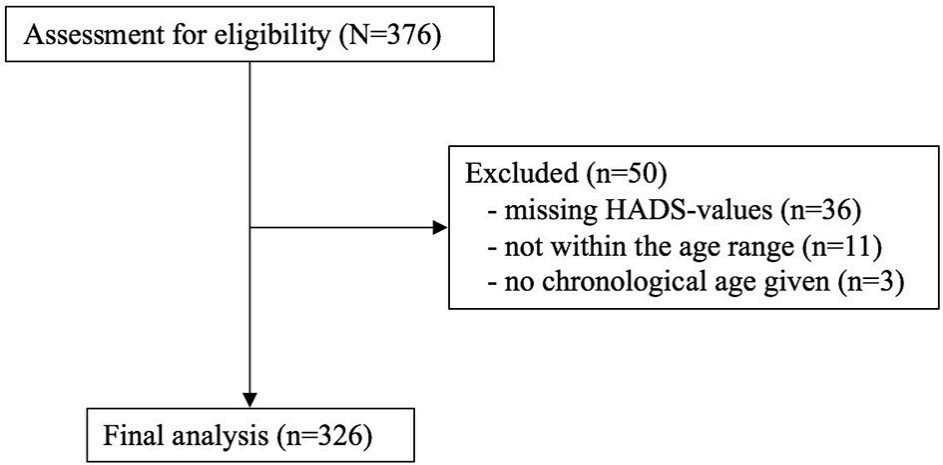
Consort flow diagram: eligibility for inclusion in the study.

Originally, 376 athletes were asked to complete the HAD Scale. Thirty-six athletes were excluded from the analysis because of missing values in the questionnaire. In addition, 14 athletes were excluded because they were not within the age range of 12–18 years or they did not provide their chronological age on the questionnaire (Figure 1).

For the distribution analysis, percentiles ranging from 50 to 98% were calculated and contrasted with cut-offs reported in the literature (Zigmond and Snaith, 1983; White et al., 1999). Furthermore, we z-transformed HAD Scale-data for anxiety and depression using the following equation:

$$\begin{array}{l} \text{Z}-\text{Score}=\left({\text{single}}_{\text{young athlete}}-{\text{mean}}_{\text{young athletes}}\right)/{\text{SD}}_{\text{young athletes}}. \end{array}$$

Z-scores above “0” indicate higher values on the HAD Scale-subscale compared to the sample mean. Z-score values below “0” indicate lower values on the HAD Scale-subscale compared to the sample mean (Dancey and Reidy, 2014). After having completed Z-transformation, it is legitimate to compare a single athlete with the group mean of all investigated athletes because the 95% confidence interval (CI) of a standard normal distribution is defined as follows: 95% CI = mean_youngathletes_ ± 1.96SD_youngathletes_ (Dancey and Reidy, 2014). *Z*-values above +1.96 were considered as relevant for diagnostic purposes (Magerl et al., 2010; Puta et al., 2013).

To detect age effects on the anxiety and depression subscales, age groups were classified based on Granacher et al. (2016) as follows: late childhood (12–14 years) and late adolescents (15–18 years). Spearman's rho correlation coefficient was used for the assessment of the relationship between HADS-A and HADS-D. All statistical analyses were carried out using SPSS Statistics Version 23. In general, graphics were made using R Software 3.3.2 with the packages ggplot2 and grid amongst other things (The R Foundation for Statistical Computing). The line of best fit for the Z-score was computed using the Local Polynomial Regression Fitting (Cleveland et al., 1992).

## Results

### Distribution of symptoms of anxiety and depression in young athletes

**Figure 2:**
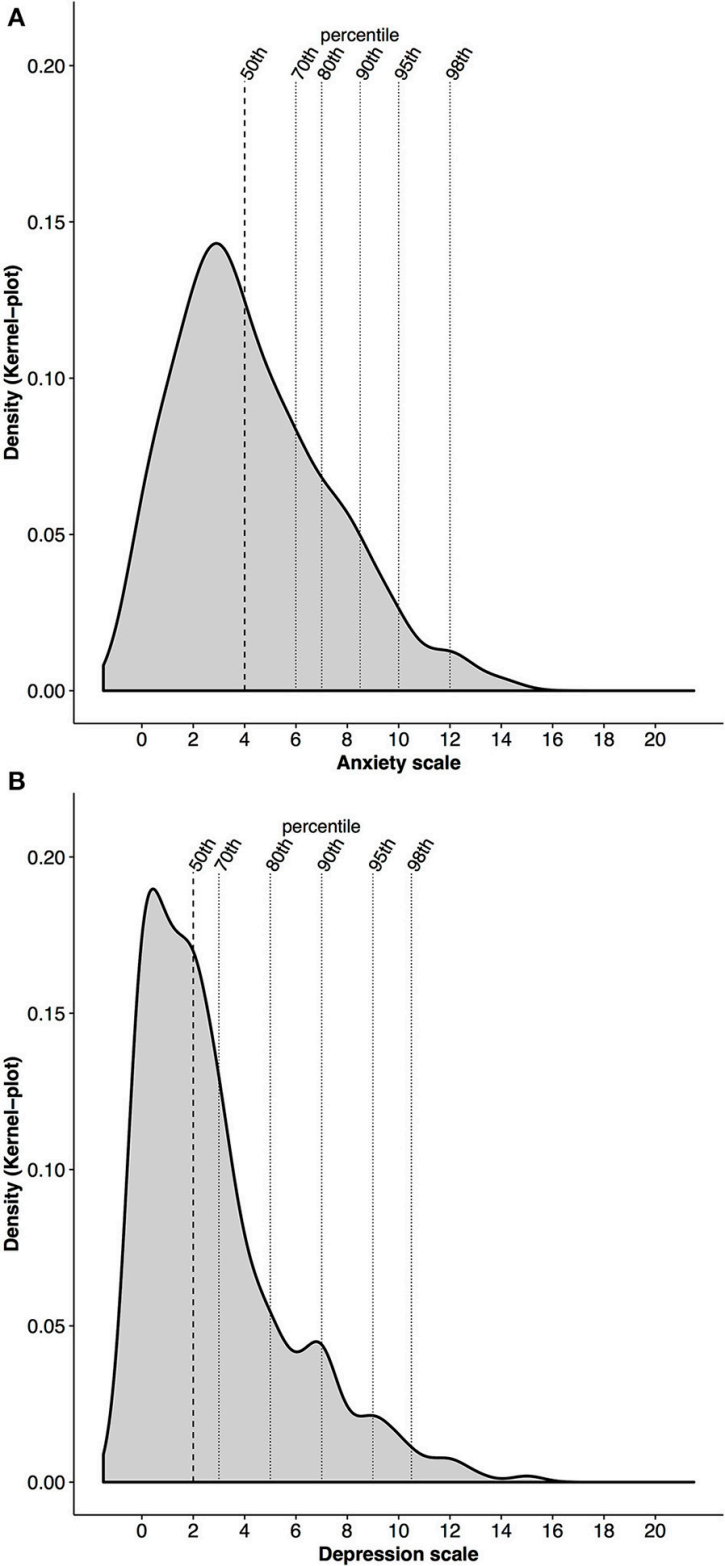
**(A,B)** Density of the distribution of anxiety **(A)** and depression **(B)** with percentiles (50th, 70th, 80th, 90th, 95th, and 98th) in alignment with the HAD Scale scores.

Both HAD Scales were not normally distributed. As indicated in Figure 2A, the anxiety scores are positively skewed (0.74) with a shift of the distribution curve to the left. The curve has a steep incline and peaks at score 3 after which it falls again. The curve declines until score 15 on the anxiety scale. Overall the minimum and maximum score for anxiety are 0 and 14, respectively (mean ± SD: 4.3 ± 3.0, Table 2). Fifty percent of the young athletes are at or below an anxiety score of 4. Furthermore, 70% of the young athletes are within a score range of 6, 80% are at or below a score of 7, 90% of athletes are at or below a score of 8.5, 95% are at or below a score of 10, and 98% of the athletes are at or below a score of 12 on the anxiety scale.

**Table 2:** Overall results (mean±SD, 95% Confidence interval of the mean) of anxiety (HADS-A) and depression (HADS-D) as well as comparison between age groups (12–14: late childhood; 15–18: late adolescents) and sex.

	**Male/Female**	**HADS-A (95% CI)**	**HADS-D (95% CI)**
**Overall**	326 (171/155)	4.3 ± 3.0 (4.01–4.67)	2.8 ± 2.9 (2.52–3.15)
**Age-group**			
12–14	88/92	4.2 ± 3.2 (3.71–4.65)	2.8 ± 3.0 (2.40–3.29)
15–18	83/63	4.5 ± 2.8 (4.08–5.00)	2.8 ± 2.7 (2.82–3.26)
**Sex**	Male: 171	4.1 ± 2.9 (3.65–4.53)	3.0 ± 2.9 (2.51–3.38)
	Female:155	4.6 ± 3.1 (4.13–5.11)	2.7 ± 2.9 (2.25–3.16)

The distribution of the depression scores is positively skewed as well (1.36). The depression curve (Figure 2B) displays a sharp incline until point 1 on the depression scale where it peaks. This is followed by a similar sharp decline until point 6 on the depression scale. After a small rise in density at point 7, the distribution of the depression scores levels off until score 16. Overall, the depression scores range from 0 to 15 (mean ± SD: 2.8 ± 2.9, Table 2). Of these, 50% of the young athletes score 2 or lower on the depression scale. Seventy percent are within a score of 3, 80% reach a score of 5, 90% a score of 7, 95% a score of 9, and 98% a score of 10.5 on the depression scale.

**Figure 3:**
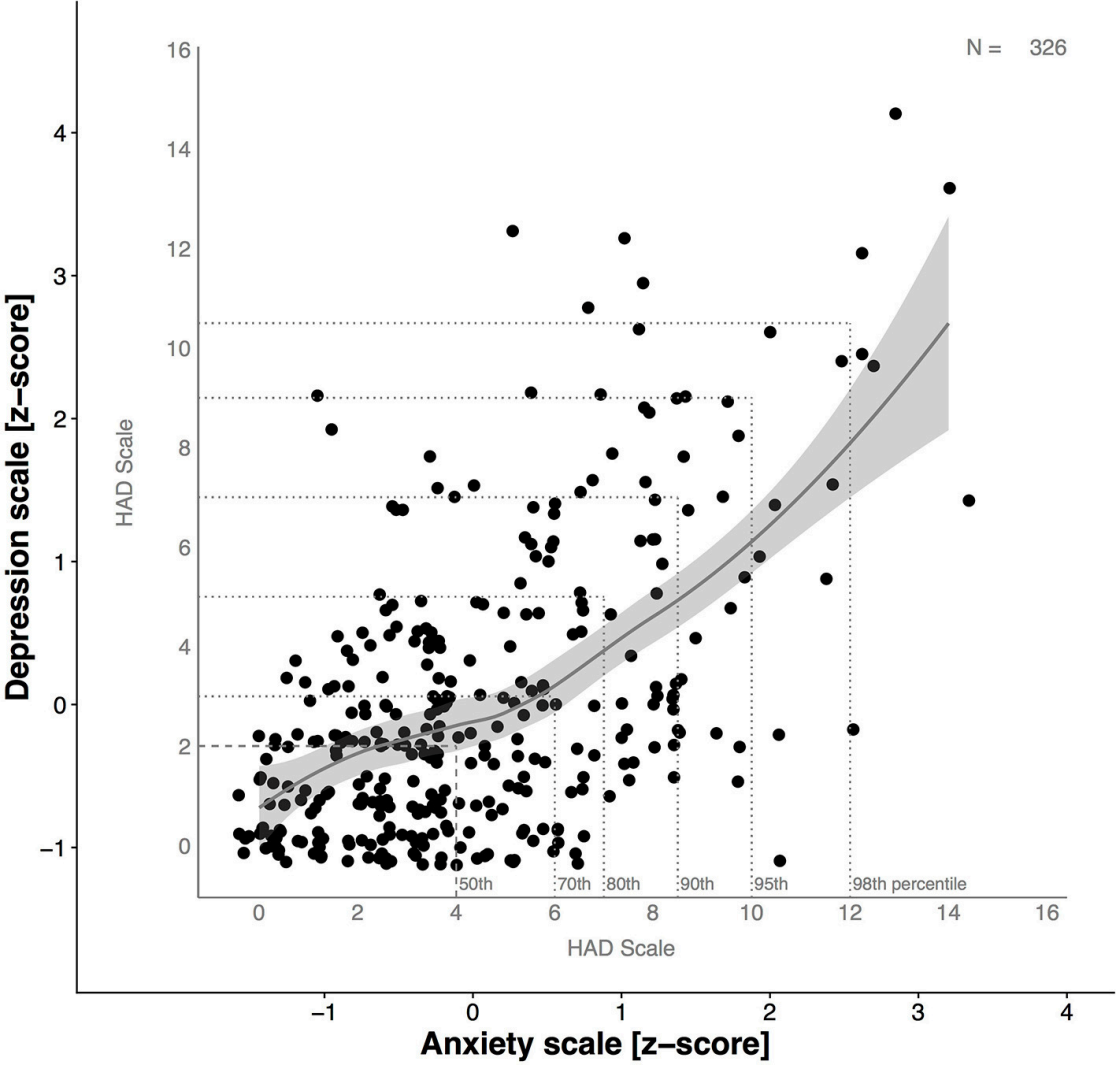
Relationship between anxiety and depression and expression as Z-scores. Line of best fit with 95% CI as highlighted in gray. Z-scores above “0” indicate higher values on the HAD Scale-subscale compared to the sample mean. Z-score values below “0” indicate lower values on the HAD Scale-subscale compared to the sample mean. *Z*-values above +1.96 were considered as relevant for diagnostic purposes. Please note: the graphic shows a slightly greater increase in anxiety than depression.

Regarding the relationship between anxiety and depression, the graphical presentation of the z-scores (Figure 3) indicates that with an increase in anxiety scores there is a concomitant increase in depression scores. However, this is not linear as can be seen from the percentiles of the distribution. For example, whilst 50% of the anxiety scores are at 4 on the anxiety scale, 50% of the depression scores are at 2 on the depression scale. This is equal to a z-score below 0 for both HAD Scales. Further, 70% of the athletes are at score 6 on the anxiety scale and 3 on the depression scale. This pattern continues until 98% of the distribution of scores (anxiety at score 12, depression at score 10.5). Furthermore, the illustration of the z-scores shows that the highest anxiety score (14) is 3 standard deviations above the mean HADS-A and the highest depression score (15) 4 standard deviations above the mean HADS-D, which highlights the spread of the data.

The combination of Z-score data together with percentiles of distribution enables a descriptive evaluation of our data, especially of the abnormal values. This is particularly useful because cut-off values vary between studies. However, when looking at the 95th percentile on the anxiety scale, it corresponds to a Z-score just below two. This however, is not the case for the depression scale where the 95th percentile is just above a Z-score of 2.

Combining the relationship of the percentiles with the scores for HADS-A and HADS-D and the distribution of the data, the Spearman rho correlation coefficient between anxiety and depression amounted to r_s_ = 0.48 (*p* < 0.01).

### Severity of symptoms of anxiety and depression in young athletes

When looking at the severity of symptoms of anxiety and depression, different methods can be used for categorization purposes. If original cut-off scores are applied, 43 (13.2%) and 11 (3.4%) of the young athletes are to be classified as doubtful cases and cases for anxiety, respectively. For depression, 18 (5.5%) and 7 (2.1%) young athletes can be classified as doubtful cases and cases, respectively (Table 3). However, when cut-off scores, as validated for the adolescent population are used, the classifications are slightly different. For HADS-A 23 (7.1%) and 10 (3.1%) young athletes can be categorized as possible and probable cases, respectively. In addition, on the depression scale 31 (9.5%) and 12 (3.7%) would be classified as possible and probable cases, respectively. Of these, 8 athletes showed subclinical values on both subscales and 6 athletes reported clinically relevant values for symptoms of anxiety and depression.

**Table 3:** Identified cases as well as percentage for anxiety (HADS-A) and depression (HADS-D) depending on cut-off levels as reported by the literature.

**Zigmond and Snaith**, **1983**				**White et al.**, **1999**		
**HADS-D**	**No case (0–7)**	**Doubtful case (8–10)**	**Case (11–21)**	**No case (0–6)**	**Possible case (7–9)**	**Probable case (10–21)**
12–14	164 (91.1%)	12 (6.7%)	4 (2.2%)	155 (86.1%)	17 (9.4%)	8 (4.4%)
15–18	137 (93.8%)	6 (4.1%)	3 (2.1%)	128 (87.7%)	14 (9.6%)	4 (2.7%)
Overall	301 (92.3%)	18 (5.5%)	7 (2.1%)	283 (86.8%)	31 (9.5%)	12 (3.7%)
**HADS-A**	**(0–7)**	**(8–10)**	**(11–21)**	**(0–8)**	**(9–11)**	**(12–21)**
12–14	149 (82.8%)	22 (12.8%)	8 (4.4%)	160 (88.9%)	12 (6.7%)	8 (4.4%)
15–18	123 (84.2%)	20 (13.7%)	3 (2.1%)	133 (91.1%)	10 (6.8%)	3 (2.1%)
Overall	272 (83.4%)	43 (13.2%)	11 (3.4%)	293 (89.9%)	23 (7.1%)	10 (3.1%)

*Overall results and depending on age group (12–14: late childhood; 15–18: late adolescents)*.

### Age and sex differences regarding the severity of symptoms of anxiety and depression in young athletes

**Figure 4:**
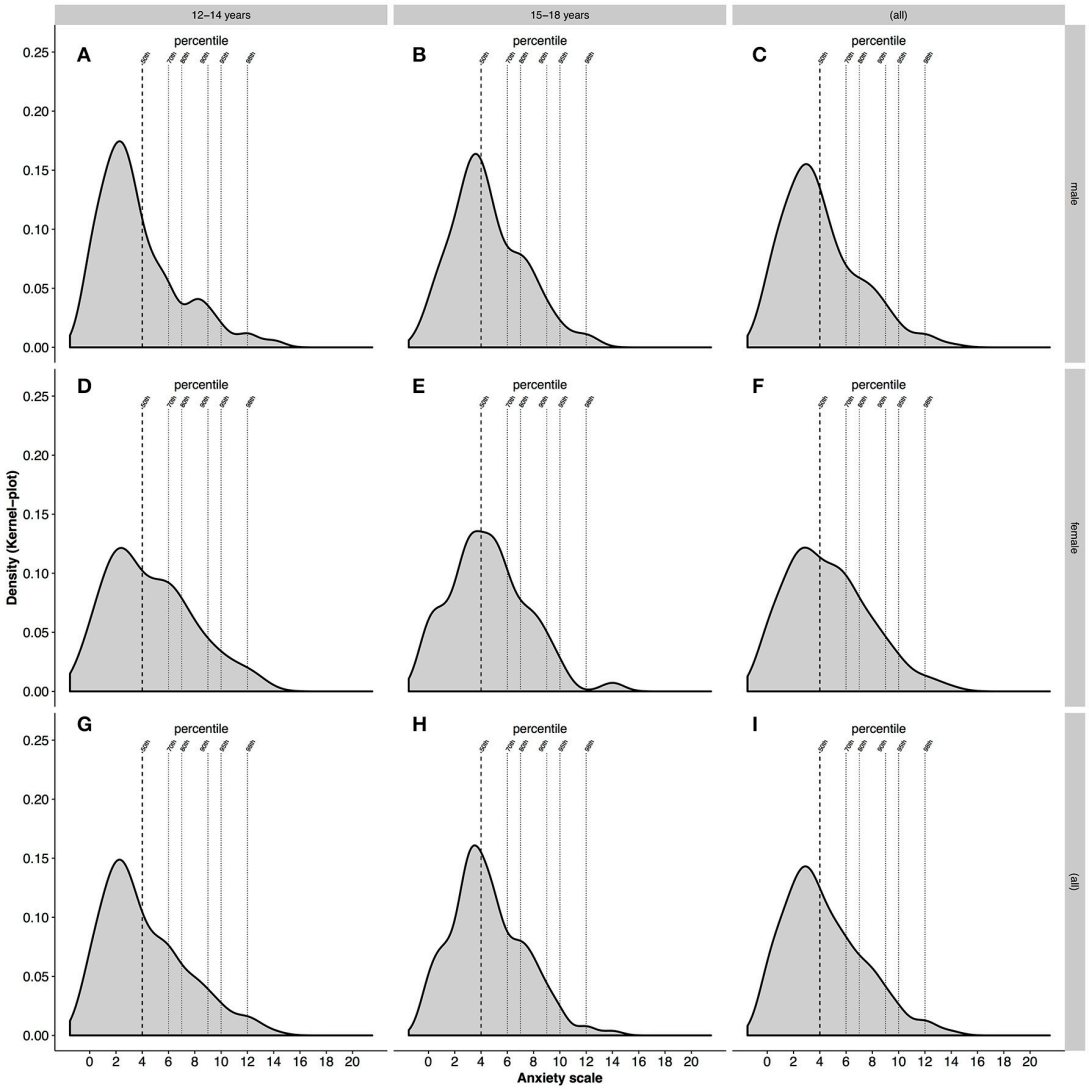
Density of the distribution of anxiety. **(A–C)** Shows male athletes in late childhood, late adolescence and overall, respectively. **(D–F)** Shows female athletes in late childhood, late adolescents and overall, respectively. **(G,H)** Shows late childhood and late adolescents regardless of gender. **(I)** Shows the density of anxiety for all athletes.

**Figure 5:**
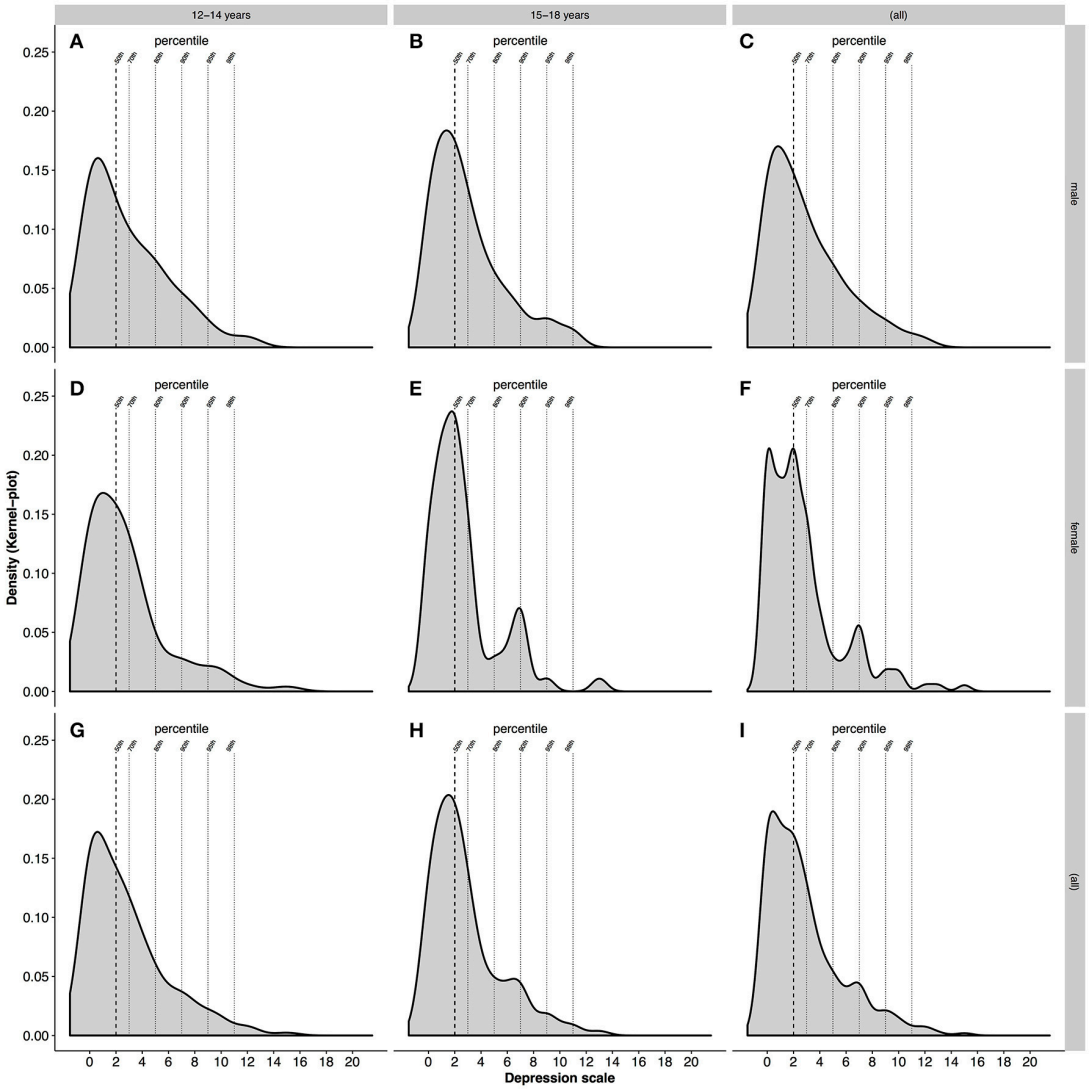
Density of the distribution of depression. **(A–C)** Shows male athletes in late childhood, late adolescents and overall, respectively. **(D–F)** Shows female athletes in late childhood, late adolescents and overall, respectively. **(G**,**H)** Shows late childhood and late adolescents regardless of gender. **(I)** Shows the density of depression for all athletes.

Age: Overall, our findings revealed that by trend (Mann-Whitney-U Test, *p* = 0.07), late childhood athletes had a slightly lower mean anxiety score (4.2 ± 3.2) than late adolescent athletes (4.5 ± 2.8). The mean of HADS-D for both age groups were the same (Table 2) with no significant between-group differences (Mann-Whitney-U Test, *p* = 0.55). In addition, both HAD Scales showed a positive skewness with regards to late childhood (HADS-A:0.9; HADS-D:1.3) and late adolescents (HADS-A:0.6; HADS-D:1.4), with a shift of the distribution curve to the left. This is illustrated when examining the distribution of scores for anxiety and depression (Figures 4, 5). For late childhood, 50% of all athletes had an anxiety score of 4 and a depression score of 2. Both distribution curves are similar, with a sharp increase in distribution and a smaller, steadier decrease (Figures 4G, 5G). Seventy percent of participants in the late childhood group had an anxiety score of 6 and a depression score of 3. At 90% of the distribution of all scores, HADS-A is at score 9 and HADS-D at score 7. Furthermore, 98% are within a score range of 12 for HADS-A and 11 for HADS-D. A similar frequency distribution is visible for athletes in the late adolescents group (Figures 4H, 5H). Whilst percentiles and HAD Scale score are the same, the distribution frequency are slightly different. For example, at 50% HADS-D the density is above 0.2, whereas for HADS-A it is only slightly above 0.15. In addition, the density for 70% of the scores at score 3 HADS-D (0.15) is higher than 70% of distribution on the anxiety scale (<0.1). Similar frequencies between HADS-A and HADS-D are reached only at 90% of distribution for late adolescence group. In terms of cut-off values for severity of symptoms of anxiety and depression further information can be found in Table 3.

Sex: As illustrated in Table 4, female athletes showed a higher mean anxiety score but a lower mean depression score than male athletes. However, the Mann-Whitney-U Test indicated no significant differences between male and female athletes for the HADS-A (*p* = 0.10) and the HADS-D (*p* = 0.46). Regarding distribution, both HAD Scales were not normally distributed. HADS-A was positively skewed for males (0.9) and females (0.6) with a shift of the distribution curve to the left. HADS-D was also positively skewed for both genders (males:1.1; females: 1.7).

**Table 4:** Identified cases as well as percentage for anxiety (HADS-A) and depression (HADS-D) depending on cut-off levels as reported by the literature and depending on sex.

**Zigmond and Snaith**, **1983**				**White et al.**, **1999**		
**HADS-D**	**No case (0–7)**	**Doubtful case (8–10)**	**Case (11–21)**	**No case (0–6)**	**Possible case (7–9)**	**Probable case (10–21)**
Male	156 (91.2%)	11 (6.4%)	4 (2.3%)	148 (86.5%)	17 (9.9%)	6 (3.5%)
Female	145 (93.5%)	7 (4.5%)	3 (1.9%)	135 (87.1%)	14 (9.0%)	6 (3.9%)
**HADS-A**	**(0–7)**	**(8–10)**	**(11–21)**	**(0–8)**	**(9–11)**	**(12–21)**
Male	146 (85.4%)	20 (11.7%)	5 (2.9%)	156 (91.2%)	10 (5.8%)	5 (2.9%)
Female	126 (81.3%)	23 (14.8%)	6 (3.9%)	137 (88.4%)	13 (8.4%)	5 (3.2%)

When looking at the density of the distribution of anxiety and depression for male and female athletes, differences in the frequency of scores are detectable (Figures 4, 5). Male young athletes show a higher frequency (>0.15) of anxiety scores below or at 4 than female athletes (<0.15), which is equal to 50% for both genders (Figures 4C,F). Therefore, a larger proportion of male compared with female athletes are below the 50th percentile of all scores. The decline of frequencies above score 4 is greater in male than female athletes. This is illustrated by a greater density of scores for female athletes at the 70th, 80th, and 95th percentile, compared with male athletes (Figure 4). Regarding the depression scores, female athletes show a greater frequency (>0.2) of scores below or at 2 on the depression scale, which is equal to 50% of all scores for both genders (Figures 5C,F). The decline in frequency distribution is steeper in female athletes. This can be seen by the distribution of scores at the 80th percentile. However, whilst the scores for depression decrease steadily in male athletes, female athletes show a higher density (>0.05) at the 90th percentile and a lower density at the 98th percentile (Figure 5).

Overall, the lowest score for anxiety and depression was 0 for male and female athletes. The highest anxiety score was 14 for both male and female athletes, whereas the highest depression score was 12 for male and 15 for female athletes.

Regarding the severity of symptoms for anxiety and depression, more male athletes had subclinical scores for depression, whereas more female athletes showed subclinical scores for anxiety. In addition, female athletes had more clinical values for anxiety, whereas no statistically significant differences were found for depression, irrespective of the classification method (Table 4).

## Discussion

The aim of this cross-sectional study was to provide an overview of the general presence and severity of symptoms of anxiety and depression in young athletes as well as to examine potential age and sex differences. The first part of the discussion refers to the aspects of distribution of symptoms of anxiety and depression, their severity, and the observed age and sex differences. This is followed by an elaboration on future implications and limitations.

### Distribution of symptoms of anxiety and depression in young athletes

The distribution of anxiety and depression scores showed that 80% of young athletes are at or below an anxiety score of 7 and at or below a depression score of 5 on the HAD Scale. However, the remaining 20% showed anxiety and depression scores that are partially above subclinical and clinical relevant scores according to both Zigmond and Snaith (1983) and White et al. (1999). It has been argued that student athletes are at higher risk of developing anxiety and depression because of higher psychological, physical, and social demands compared to their non-athletic peers (Wylleman et al., 2004). Despite this, the overall anxiety and depression scores of the present study are lower compared to normative data reported for adolescents in Sweden, China, and Great Britain (White et al., 1999; Jörngården et al., 2006; Chan et al., 2010). In these studies, mean anxiety scores of up to 7.2 and mean depression scores of 5.4 were reported (White et al., 1999; Jörngården et al., 2006). However, these studies lack information on sport participation of the examined subjects. Furthermore, most of these studies did not report the frequencies of scores or how many possible or probable cases of anxiety or depression were found. The observed methodological inconsistencies between our study and the studies reported in the literature make it difficult to compare the study findings.

Compared to the existing literature, the present study demonstrated, that whilst mean anxiety or depression scores might seem low, there are participants that do report high scores on one or both subscales which is indicative for the need of individual analyses. In addition, there was a non-significant tendency toward higher anxiety than depression scores as seen by the z-score distribution. Longitudinal and cross-sectional studies found a positive relationship in the occurrence of anxiety and depression, in which it seems that anxiety precedes depression and the onset of depression is more likely in individuals with higher anxiety frequencies and is independent of age of the onset of anxiety (Beesdo et al., 2009). This could explain the shift toward higher anxiety than depression scores by individuals. However, this also indicates that young athletes with high anxiety scores are possibly at risk of developing depression as well.

At this point, we would like to suggest, that the use of percentiles in combination with the Z-score distribution might be another way of analyzing symptoms of anxiety and depression using the HAD Scale. Our findings clearly showed that the 95th percentile and the Z-score are equal on the anxiety scale (10). This again corresponds to the cut-off point for possible cases (Zigmond and Snaith, 1983). In addition, Figure 3 shows that Z-scores are well-suited to highlight and identify abnormal values.

### Severity of symptoms of anxiety and depression in young athletes

In the present study, subclinical and clinical scores of anxiety and depression in young athletes were detected. Previous research used different methodological approaches to examine severity for anxiety and depression. Both Zigmond and Snaith (1983) as well as White et al. (1999) validated the HAD Scale screening properties. Whilst the former tested it on an adult population, the later verified the validity for the use in adolescent populations. When comparing identified cases for the young athletic population more subclinical and clinically relevant cases for HADS-D were identified using White et al. (1999) cut-off values. However, for HADS-A less subclinical and clinically relevant cases were detected. White et al. (1999) used lower cut-off values for depression than for anxiety, arguing that lower cut-offs may minimize the risk of false negative diagnostics for depression and a higher cut-off for anxiety may minimize the risk of false positive diagnostics. This could by why higher cases for HADS-D and the lower cases for HADS-A of the present study.

Overall, the high frequencies and severities of anxiety and depression in adolescents might be explained by the transition phase “adolescents” which the athletes are confronted with and go through. As mentioned previously, young athletes are faced with changes on various levels in their life. These may lead to an imbalance in their emotional homeostasis and therefore to the development of anxiety or depression (Spear, 2000). Regardless of this, we would like to emphasize that our analyses identified athletes with subclinically or clinically relevant symptoms in both HAD Scale subscales. It is recommended to monitor and accompany these athletes over a longer period of time to elucidate whether these symptoms are acute or chronic. As an effective means, relaxation or stress management techniques could be introduced (for example: https://medium.com/@kingsstudy/prevention-of-psychological-stress-in-youth-athletes-2c086bbf7640) to provide easy-to- administer but effective instruments for these athletes.

### Age and sex differences regarding the severity of symptoms of anxiety and depression in young athletes

In the present study, no significant differences were found between the age groups and symptoms of anxiety and depression. This is in contrast to previous research, that showed an increase in emotional distress between 13 and 15 years, 16 and 19 years, and 20 and 23 years (Jörngården et al., 2006). However, a possible explanation for this discrepancy in findings might be that anxiety and depression disorders tend to naturally grow and decline over time in a young age group (Beesdo et al., 2009). In addition, since this is a cross-sectional study, it can only give a momentary analytic view of symptoms of anxiety and depression. Only long-term analysis could confirm possible changes that occur during the different age phases of adolescents.

With regards to sex, no differences were found between anxiety and depression, although female adolescent athletes scored higher in both subscales of the HAD Scale. These findings are in contrast with previous research. Studies using the HAD Scale on the adolescent population found sex differences in the subscales anxiety and depression. White et al. (1999) reported significant higher anxiety and depression scores for female compared to male adolescents. Jörngården et al. (2006) observed higher anxiety scores for females but no sex differences in depression scores. Brand et al. (2013) reported that female adolescent athletes showed more frequently symptoms of anxiety and depression than their male peers. In addition, Chan et al. (2010) reported significantly higher anxiety scores for female compared to male adolescents but higher depression scores for male compared to female adolescents. This is of interest for results of the present study, although not significant, similar results were found. Female young athletes had higher mean anxiety scores, which was related to more subclinical and clinical cases. In contrast, male young athletes showed higher mean depression scores, which was accompanied by more subclinical cases than in female athletes. This implies a tendency toward sex differences, especially on a subclinical scale.

Potentially underlying reasons for the observed higher anxiety scores in females compared with males might be that male adolescents tend to be more confident, more open to contact with others (peers), and need less approval of others, whereas females tend to have higher levels of worry and lower levels of self-esteem (Byrne, 2000; Grossbard et al., 2009; Falgares et al., 2017). Of note, low self-esteem and self-blame have been found to be significant predictors of depressive and anxiety symptoms in adolescents (Garnefski et al., 2002).

### Future implications and limitations

#### Future implications

Anxiety can be defined as “a state of anticipatory apprehension over possible deleterious happenings” and “involves anticipatory affective arousal that is cognitively labeled as state of fright” (Bandura, 1988, p. 77). The process of arousal is affected by the balance between perceived coping capabilities and probable hurtful aspects of the environment (Bandura, 1988). Anxious states are accompanied by subjective distress and corresponding physiological changes in heart rate and catecholamine secretion (Bandura et al., 1985; Sanchez-Gonzalez et al., 2015). Risk factors for the development of anxiety are diverse and range from childhood abuse, neglect, or violence to low socio-economic status, female gender, and an intolerance to uncertainty (Stein and Sareen, 2015). In addition, persons with high levels of anxiety are at risk of developing depression and deliberate self-harm (Frances et al., 1992; Stein and Sareen, 2015).

Depression can be described as a state of having a negative view of the world, oneself and the future, as well as having a lack of interest with anhedonia and reduced energy (Willner et al., 2013; Belzug et al., 2015). There are various causes for the development of depression for instance early environmental factors such as lack of emotional contact with parents, traumatic experiences, or poor quality of parental care. All these may lead to low self-esteem and emotional instability (Willner et al., 2013).

In the present study, many of the described risk factors such as socio-economic status or parental relationship have not been explicitly investigated. Therefore, one cannot conclude that the high scores of anxiety and depression were caused by these risk factors, nor can one assume that the tight schedule of daily training, school, and competition are underlying causes. It may be that a combination of different factors leads to high anxiety and depression scores in individual young athletes (Brettschneider, 1999; Merkel, 2013). Whilst the described risk factors are associated with the environment, a psychodynamic approach is concerned with interpersonal dynamics as well as life experiences that may lead to psychological maladaptation. This developmental approach deals with the process of self-definition and relatedness during adolescents and throughout life (Blatt and Luyten, 2009). Self-definition refers to the development of realistic, integrated and differentiated identity or sense of self. Relatedness comprises the ability of mature, intimate, reciprocal and mutually satisfactory interpersonal relationships. These two processes are equally important and work in a synergistic manner (Blatt and Luyten, 2009). Research shows that stability and quality of friendships during adolescents are positively related to the development of sense of self and lower levels of depressive symptoms (Kopala-Sibley et al., 2016). In addition, abnormal self-definition (self-criticism) and relatedness (dependency) are associated with anxiety and suicidality (Falgares et al., 2017). The feeling of self-criticism and dependency is accompanied by the feeling of hopelessness and affective temperament expression, which again is related to anxiety, depression and even suicidal behavior (Nkansah-Amankra et al., 2010; Iliceto et al., 2011).

What are the most relevant implications of the present study? How should legal representatives, coaches, and teachers act regarding the frequency of reported subclinical and clinically relevant scores?

Research shows that psychological problems often only mature in mid to late adolescents (Blatt and Luyten, 2009). Therefore, the high prevalence of subclinical and clinically relevant scores call for the need of intervention strategies that aid in the prevention of the development of psychological distress, as well as aid with already existing problems. Since environmental factors like parental relationship or socio-economic status cannot be controlled, one should focus on the teaching of coping strategies, self-efficacy, mindfulness, and stress reduction techniques to prevent the development of psychological symptoms and thereby teaching trust in one's self and one's ability to manage stressful situations. Research shows a reduction in anxiety and perceived stress through mindfulness, relaxation, and stress reducing techniques in the adolescent population (Foret et al., 2012). In addition, the implementation of coping strategies for adolescent athletes were found to increase self-efficacy (Reeves et al., 2011). Exercising self-efficacy can help reduce anxiety and increase optimism and hope for success (Bandura, 1988; Zagórska and Guszkowska, 2014).

Furthermore, research shows that young athletes might not be aware of the symptoms of psychological distress or where to seek help. In addition, young athletes are confronted with barriers that could stop them form help seeking. These include, worry about affecting ability to train, fearing what might happen, not knowing who to ask or lack of time (Gulliver et al., 2012). Therefore, it seems necessary to teach young athletes the awareness of psychological symptoms as well as introducing aforementioned techniques into the daily routine of young athletes (e.g., https://medium.com/@kingsstudy/prevention-of-psychological-stress-in-youth-athletes-2c086bbf7640). In addition, athletes should be given the possibility to seek help by a psychologist if needed.

#### Limitations

This study has various limitations that warrant discussion because they have not been considered or examined and may have therefore restricted the view on our results. There are several factors within the sporting context that might be related to the occurrences of symptoms of anxiety and depression such as injury status, playing position, and individual vs. team sport participation (Wolanin et al., 2015; Nixdorf et al., 2016; Prinz et al., 2016). Further, socio-economic status as well as parental care and relationship can affect the development of psychological symptoms (Beesdo et al., 2009; Willner et al., 2013; Stein and Sareen, 2015). In addition, the present study shows a cross-sectional view of the young athletes' population. However, the level of anxiety amongst adolescents tends to fluctuate over time (Beesdo et al., 2009). Therefore, identified cases may also fluctuate over time. Moreover, the HAD Scale relies on self-reported measurements, which are susceptible to bias. Research shows that prevalence rates of depression differ depending on the diagnostic instrument used. Whilst questionnaires can quantify severity and identify possible changes over time, structured clinical interviews are the gold standard for identifying clinical significance and potential treatment (Trask, 2004; Krebber et al., 2014). In addition, the HAD Scale investigates symptoms over the last 2 weeks, thereby limiting possible assessment of long-term psychological state of the young athlete. Therefore, further research should examine possible long-term changes of symptoms of anxiety and depression in adolescent athletes. Moreover, identified subclinical and clinical cases would have to be investigated further using structured clinical interviews.

## Conclusion

To the best of our knowledge, this is the first study that examined distribution and prevalence rates of symptoms of anxiety and depression in young athletes with a focus on detecting clinically relevant findings. Our results show prevalence rates of up to 9.5% on a subclinical scale and up to 3.7% on a clinical scale. Moreover, we detected that some young athletes are at risk of developing symptoms of both anxiety and depression. However, our findings cannot be interpreted that there is a general risk in developing psychological disorders particularly in young athletes. The longitudinal and case based assessment of symptoms of anxiety and depression in young athletes might provide more information and insights over the long-term persistence and possible underlying causes of the symptoms.

## Author contributions

CP, UG, and ML designed the experiment. ML and BG gathered data. SW, HG, and CP conducted data analysis. SW, CP, and HG wrote the manuscript. HG, CP, TS, and SW conducted graphical representation, K-JB and MH assisted with possible clinical psychological questions. All authors discussed the results and its implications, commented and edited the manuscript at all stages, and approved the final version.

### Conflict of interest statement

The authors declare that the research was conducted in the absence of any commercial or financial relationships that could be construed as a potential conflict of interest.
